# Uptake and Presentation of Myelin Basic Protein by Normal Human B Cells

**DOI:** 10.1371/journal.pone.0113388

**Published:** 2014-11-17

**Authors:** Marie Klinge Brimnes, Bjarke Endel Hansen, Leif Kofoed Nielsen, Morten Hanefeld Dziegiel, Claus Henrik Nielsen

**Affiliations:** 1 Institute for Inflammation Research, Department of Infectious Diseases and Rheumatology, section 7521, Copenhagen University Hospital Rigshospitalet, Copenhagen, Denmark; 2 Immudex, Copenhagen, Denmark; 3 Department of Technology, Faculty of Health and Technology, Metropolitan University College, Copenhagen, Denmark; 4 Blood Bank, KI2034, Department of Clinical Immunology, Copenhagen University Hospital Rigshospitalet, Copenhagen, Denmark; University of Texas at San Antonio, United States of America

## Abstract

B cells may play both pathogenic and protective roles in T-cell mediated autoimmune diseases such as multiple sclerosis (MS). These functions relate to the ability of B cells to bind and present antigens. Under serum-free conditions we observed that 3–4% of circulating B cells from healthy donors were capable of binding the MS-associated self-antigen myelin basic protein (MBP) and of presenting the immunodominant peptide MBP85-99, as determined by staining with the mAb MK16 recognising the peptide presented by HLA-DR15-positive cells. In the presence of serum, however, the majority of B cells bound MBP in a complement-dependent manner, and almost half of the B cells became engaged in presentation of MBP85-99. Even though complement receptor 1 (CR1, CD35) and CR2 (CD21) both contributed to binding of MBP to B cells, only CR2 was important for the subsequent presentation of MBP85-99. A high proportion of MBP85-99 presenting B cells expressed CD27, and showed increased expression of CD86 compared to non-presenting B cells. MBP-pulsed B cells induced a low frequency of IL-10-producing CD4+ T cells in 3 out of 6 donors, indicating an immunoregulatory role of B cells presenting MBP-derived peptides. The mechanisms described here refute the general assumption that B-cell presentation of self-antigens requires uptake via specific B-cell receptors, and may be important for maintenance of tolerance as well as for driving T-cell responses in autoimmune diseases.

## Introduction

In addition to producing antibodies, B cells are highly efficient antigen-presenting cells (APCs) and produce a variety of cytokines [1]. B cells are capable of taking up small amounts of their cognate antigen and presenting it to T cells [2]. Complement receptors (CRs) may contribute to antigen uptake by B cells, either by cross linking CR2 and the B-cell receptor (BCR), or as a BCR-independent internalisation receptor [3], [4]. In contrast to antigen-specific BCRs, CRs recognise antigens coated with fragments of complement component 3 (C3) or in the context of complement-coated immune complexes [4]–[11]. CR2-mediated antigen uptake by B cells bypasses the need for antigen specificity, and increases the proportion of B cells engaging in antigen-presentation [12]. We have previously shown that CR2 contributes to B-cell binding of the self-antigen thyroglobulin, which is capable of forming immune complexes with naturally occurring or disease-associated autoantibodies [12], [13]. It is not known, however, whether CR2-dependent uptake is sufficient for presentation of self-antigens to occur. Depending on the circumstances, this could either potentiate immune responses or mediate T-cell tolerance.

Recently, much research has focused on a subset of B cells with immunoregulatory potential, known as regulatory B cells (Bregs) [14]–[17]. These B cells assist in maintaining peripheral tolerance by secreting immunoregulatory cytokines [15], [17]. The phenotypic definition of Bregs is still controversial because production of the immunomodulating cytokine interleukin-10 (IL-10) is their only hallmark [14]. Moreover, several studies have demonstrated cross-talk between Bregs and regulatory T cells (Tregs) [18]–[20] and, apart from IL-10 production [20], especially the expression of CD80 and CD86 seems important in this interaction [18], [20]. Activated B cells derived from MS patients show decreased IL-10 production [21]. Usually, polyclonal stimuli such as toll-like receptor ligands are used to stimulate human B cells to produce IL-10 (for review see [22]), but the self-antigen thyroglobulin also induces IL-10 production by approximately 1% of normal B cells [23].

Propathogenic B cells are involved in the maintenance of autoimmune diseases, as demonstrated by the beneficial effect of the B cell-depleting antibody rituximab in a number of autoimmune diseases [24]. These include relapsing-remitting multiple sclerosis (MS) [25], [26], an inflammatory, demyelinating disease of the central nervous system (CNS) characterised by an immunological attack on the myelin sheath in the CNS orchestrated by autoreactive CD4+ T cells [27]. MS is associated with the human histocompatibility leukocyte antigen (HLA)-DR15 haplotype [28], indicating that major histocompatibility complex class II-restricted presentation of CNS-derived antigens is important in the disease process. Reduced relapse rates in the first 24 weeks of B-cell depletion without a significant influence on total antibody level [25] suggest that the pathogenic role of B cells is associated with antigen-presentation [29] and secretion of pro-inflammatory cytokines [30], rather than with antibody production. B-cell numbers are elevated in the CNS in the majority of MS patients [31].

B-cell antigen presentation is usually studied by indirect measurement of the resulting T-cell response [5], [6], [10], [12], [32]. Using CD4+ T-cell activation as read-out, we and others have previously examined the ability of B cells to present self-antigens such as thyroglobulin [23] and aggrecan [33]. However, antigen presentation that leads to downregulation of T-cell responses is difficult to assess in this manner, and information about the proportion and phenotype of the B cells presenting the antigen is usually missing. Here we examine directly the B-cell uptake and presentation of the self-antigen myelin basic protein (MBP), a self-antigen considered to be involved in the pathogenesis of MS [27], exploiting the recognition of the immunodominant peptide MBP85-99 presented on HLA-DR15 by mAb MK16 [28]. We also aimed to determine the role of complement receptors in the process, and to characterise the phenotypic profile of the B cells that most efficiently present MBP85-99.

## Materials and Methods

### Cells and serum

Peripheral blood mononuclear cells (PBMCs) and serum were isolated from healthy blood donors attending the Blood Bank at Copenhagen University Hospital Rigshospitalet in tubes containing heparin or no anti-coagulant (BD Bioscience, San Jose, CA). Buffy coats or heparin blood derived from 6 HLA-DR15-positive donors were used for experiments analysing the presentation of MBP peptide. Another 25 healthy blood donors with unknown HLA-tissue type were used for i) assessing the role of complement inactivation and blockade of CR1/CR2/FcRs on surface binding of MBP, ii) verifying classical complement activation in the presence of serum and iii) in co-culture studies of B cells and T cells. The donors were anonymous to the investigators, and thus no local Ethical Committee approval was required according to Danish legislation. Gradient centrifugation over LymphoPrep (Axis-Shield, Oslo, Norway) was used to isolate PBMCs. The cells were washed twice in phosphate buffered saline (GIBCO, Invitrogen, Carlsbad, CA, USA) and were resuspended in Roswell Park Memorial Institute (RPMI) 1640 medium containing HEPES (Biological Industries Israel Beit-Haemek Ltd, Kibbutz Beit-Haemek, Israel), L-glutamine (GIBCO) and gentamicin (GIBCO). Cells were either used directly in MBP surface-binding experiments or stored in liquid nitrogen before use in MBP peptide-presentation experiments. PBMCs were labelled with 5-carboxy-2′,7′-dichlorofluorescein diacetate succinimidyl ester (CFSE) at 0.25 µM in RPMI 1640 for 10 min at 37°C. CD19+ B cells and CD3+ T cells were purified from freshly purified PBMCs using the Human B cell Enrichment Kit or CFSE-labelled PBMCs using the Human CD3 Selection Kit (StemCell Technologies Inc, Vancouver, Canada).

Unless otherwise stated, serum from blood group AB donors (Lonza, Basel, Switzerland) was used as the source of normal human serum (NHS).

### Antigens

Whole human MBP was purchased from HyTest Ltd. (Turku, Finland) and was used either unconjugated or conjugated with biotin using the LYNX rapid conjugation kit (AbD serotec, Kidlington, UK), according to the manufacturer's instructions. Tetanus toxoid (Statens Serum Institut, Copenhagen, Denmark) and thyroglobulin (Biogenesis Ltd., Poole, England) were used as control antigens.

### Antibodies

The monoclonal antibody MK16 that recognizes MBP85-99 in the context of HLA-DRB1*1501 [28] was used as probe for antigen presentation. MK16 was originally obtained by phage display technology [28] in the Fab format, and was subsequently modified into a murine IgG1 antibody expressed in Chinese hamster ovary (CHO) cells [34]. The MK16 IgG1 antibody (referred to in the following as MK16) was affinity-purified by protein A from the supernatant of the MK16-expressing CHO cells grown in HAMS F-12 media (GIBCO) supplemented with 10% fetal calf serum (FCS; Biological Industries) and 0.8 mg/ml geneticin (Invitrogen, Carlsbad, CA, USA). Murine anti-human CR1 IgG1 antibody (mAb3D9) was kindly donated by Dr John O'Shea (Frederick Cancer Research and Development Center, Frederick, MD, USA), and polyclonal sheep anti-human CR2 was purchased from R&D Systems (Minneapolis, MN, USA). Mouse antibody against human glycophorin A (GP-A, CD235a) was purchased from Beckman Coulter (Brea, CA, USA). FITC-anti-human C3 (recognising C3, C3b and iC3b) and biotin-anti-human C1q for detection of C3 and C1q deposit on B cells was purchased from LifeSpan BioSciences, Inc, Seattle, WA, USA and Abcam, Cambridge, MA, USA respectively.

For flow cytometric characterisation of B-cell and T-cell subsets, the following fluorochrome-conjugated monoclonal antibodies were used: FITC-anti-human CD3, PE-Cy7-anti-human CD4, PerCP-anti-human CD14, APC-anti-human CD19, FITC-anti-human CD19, PE-anti-human CD27, APC-anti-human CD86 and PE-Cy7-anti-human CD80 (all from BD Biosciences, San José, CA, USA).

### Proliferation assay in co-cultures of B cells and T cells

250,000 CFSE-labelled CD3+ T cells were mixed with 100,000 CD19+ B cells plus 30 µg/ml MBP. Cells were cultured in RPMI 1640 in round-bottomed 96-well plates containing 30% (v/v) NHS for 7 days at 37°C and 5% CO_2_. As positive controls, 250,000 CFSE-labelled CD3+ T cells were added to wells coated with anti-CD3 (OKT3) at a concentration of 0.5 µg/ml (eBioscience, San Diego, CA, USA). At day 7, cells were stained for expression of CD4, and proliferation was measured by flow cytometry on a FACS Calibur cytometer (BD Biosciences). Background proliferation was assessed in cultures without added MBP.

### IL-10 and TNF-alpha secretion assay

500,000 CD3+ T cells were mixed with 100,000 CD19+ B cells ±30 µg/ml MBP. Cells were cultured in RPMI 1640 in flat-bottomed 96-well plates containing 30% (v/v) autologous serum for 18 h at 37°C under 5% CO_2_. As positive control, cells were cultured with staphylococcal enterotoxin B (SEB) at a concentration of 1 µg/ml. Cultures containing only T cells were used as negative controls. Production of IL-10 and TNF-alpha were measured using MACS cytokine secretion assay for IL-10 and TNF-alpha according to the manufacturer's instructions (Miltenyi Biotec, Bergisch Gladbach, Germany). Samples containing only detection antibodies were included as controls and these values were subtracted from all other samples. B-cell preparations contained 97.7±0.47% B cells, 1.5±0.43% T cells, and 0.68±0.15% monocytes, while T-cell preparations contained 98.9±0.40% T cells, 0.92%±0.38 B cells, and 0.15±0.05% monocytes. For each sample, between 100,000 and 150,000 CD4+ T cells were recorded using a FACS Canto flow cytometer (BD Biosciences).

### Assessment of MBP deposition on B cells

0.3×10^6^ PBMCs from healthy donors were incubated for 30 min at 37°C under 5% CO_2_ in LGM-3 media (Lonza, Walkersville, MD, USA) with 30 µg/ml biotinylated MBP either in the absence of serum, or in the presence of 30% (v/v) autologous serum, 30% (v/v) heat-treated (30 min at 56°C) autologous serum, or 30% (v/v) autologous serum containing 50 mM EDTA or sodium polyanethole sulfonate (SPS) at a concentration of 0.2 mg/ml or 2 mg/ml respectively (Sigma, St Louis, MO). Cells were washed twice in PBS/2% FCS and then incubated with 0.6 µg/ml streptavidin-PE and APC-anti-human CD19 for 30 min at 4°C. Binding of MBP to B cells was measured as mean fluorescence intensity (MFI) values on the total CD19+ population. Cells were analyzed by flow cytometry using a FACS Calibur cytometer (BD Biosciences). To stain for dead cells, 7-actinomycin D (7-AAD) (BD Biosciences) was added to samples before acquisition.

### Assessment of MBP presentation

Aliquots of MK16 were conjugated with biotin using the LYNX rapid conjugation kit (AbD serotec, Kidlington, UK), according to the manufacturer's instructions, or with fluorescein isothiocyanate (FITC; Sigma-Aldrich GmbH, USA) to an FITC∶protein ratio of 6∶1.

0.5×10^6^ HLA-DR15+ PBMCs were incubated for 18 h at 37°C under 5% CO_2_ in media containing 30% v/v AB serum plus 30 µg/ml of whole MBP. In some experiments thyroglobulin and tetanus toxoid were included as controls at a final concentration of 30 µg/ml. Next, the cells were incubated with IgG for intravenous use (IVIg; CSL Behring, Bern, Switzerland) at a concentration of 6 mg/ml and 2% mouse serum (Statens Serum Institut, Copenhagen, Denmark) to block unspecific binding. Subsequently, MK16 was incubated at a concentration of 50 ng/ml for 30 min at 4°C in 2% FCS; antibodies against cell-surface markers were included in the same step. Following two washes, streptavidin-PE (BD Biosciences) was incubated with the samples for 30 min at 4°C in experiments using biotinylated MK16. Finally, cells were analysed on a FACS Canto flow cytometer (BD Biosciences). To exclude dead cells, 7-AAD was added to samples before acquisition.

### Assessment of complement deposition on B cells

0.3×10^6^ PBMCs were incubated for 5 or 15 min at 37°C in LGM-3 media ±30 µg/ml of MBP and ±30% v/v autologous serum. Afterwards the tubes were kept on ice, and cells were stained with FITC-anti-human C3, biotin-anti-human C1q, and PE-Cy7-anti-human CD19 followed by a second stain by streptavidin-PE. To exclude dead cells, 7-AAD was added to samples before acquisition. Cells were analysed on a FACS Canto flow cytometer (BD Biosciences).

### Inhibition of B-cell binding and presentation of MBP by CR1 and CR2 blockade

To inhibit uptake and presentation of MBP by B cells, 0.3×10^6^ PBMCs and 1×10^6^ PBMCs respectively were incubated for 30 min at room temperature with monoclonal anti-CR1 (clone 3D9), polyclonal anti-CR2 antibodies, or a combination of the two, each at a final concentration of 10 µg/ml. As negative control, an irrelevant antibody, anti-human glycophorin (GP)-A (CD235a) was used at a concentration of 10 µg/ml. In experiments analysing presentation of MBP, cells were incubated for 1.5 h or 4 h at 37°C with 30 µg/ml MBP in media containing 30% v/v NHS. Excess MBP was washed away, and cells were incubated for 18 h at 37°C in media containing 30% v/v NHS. Otherwise, the experiments were carried out as described above. Background MFI values from samples incubated without MBP were subtracted from all values.

### Statistics

Data was analysed using FACS Diva (BD Biosciences) or FlowJo v.X, (TreeStar, Inc, Ashland, OR, USA).

Student's paired *t*-test was used. Kolmogorov-Smirnovs test was used to test for normality. P-values<0.05 were considered statistically significant.

## Results

### Binding of MBP to normal B cells

**Figure 1:**
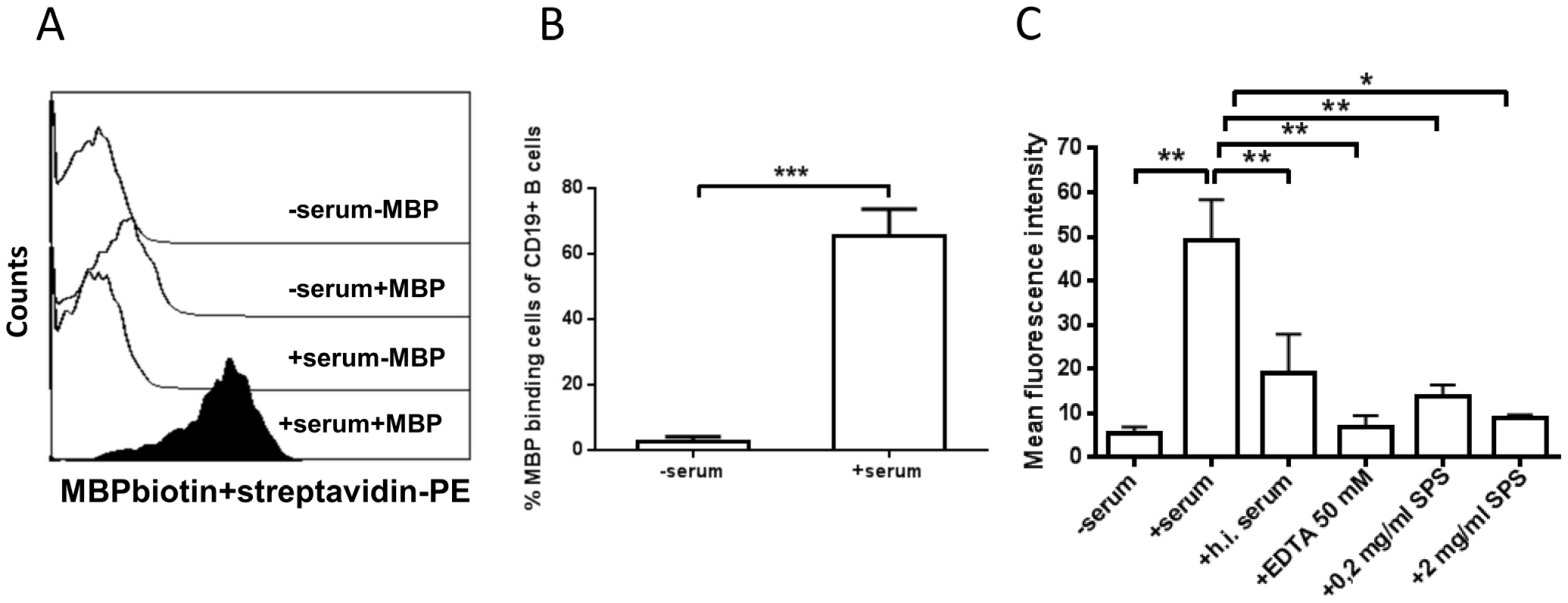
Serum complement promotes the binding of MBP to normal B cells. PBMCs from healthy donors were incubated for 30 min with or without 30 µg/ml biotinylated MBP in medium containing normal serum (30% v/v), or in pure medium. (A) Histogram plot depicting MFI values of MBP binding to B cells in one representative healthy donor. B) The binding of MBP to B cells from 7 healthy donors is shown, expressed as percentage MBP-positive B cells. C) Before addition to the culture media, serum was treated in one of three ways: heat-inactivated (h.i.) by heating to 56°C for 30 min, or supplemented with EDTA or sodium polyanethole sulphonate (SPS) in different concentrations. The resulting MFI values from 5–7 healthy donors are shown. Bars and error bars represent means and SEM *p<0.05 **p<0.01 and ***p<0.001.

We assessed the binding of MBP to B cells in cultures of normal PBMCs. In the absence of serum, little binding occurred: only 2.5±1.5% (mean±SEM) of the cells stained positive for MBP binding (Figs. 1A and B). By contrast, addition of autologous serum to the medium resulted in a shift of the entire B-cell population towards higher MBP binding. Under these conditions, 65.4±8.2% of the cells had MFI values above the negative control (no addition of MBP).

**Figure 2:**
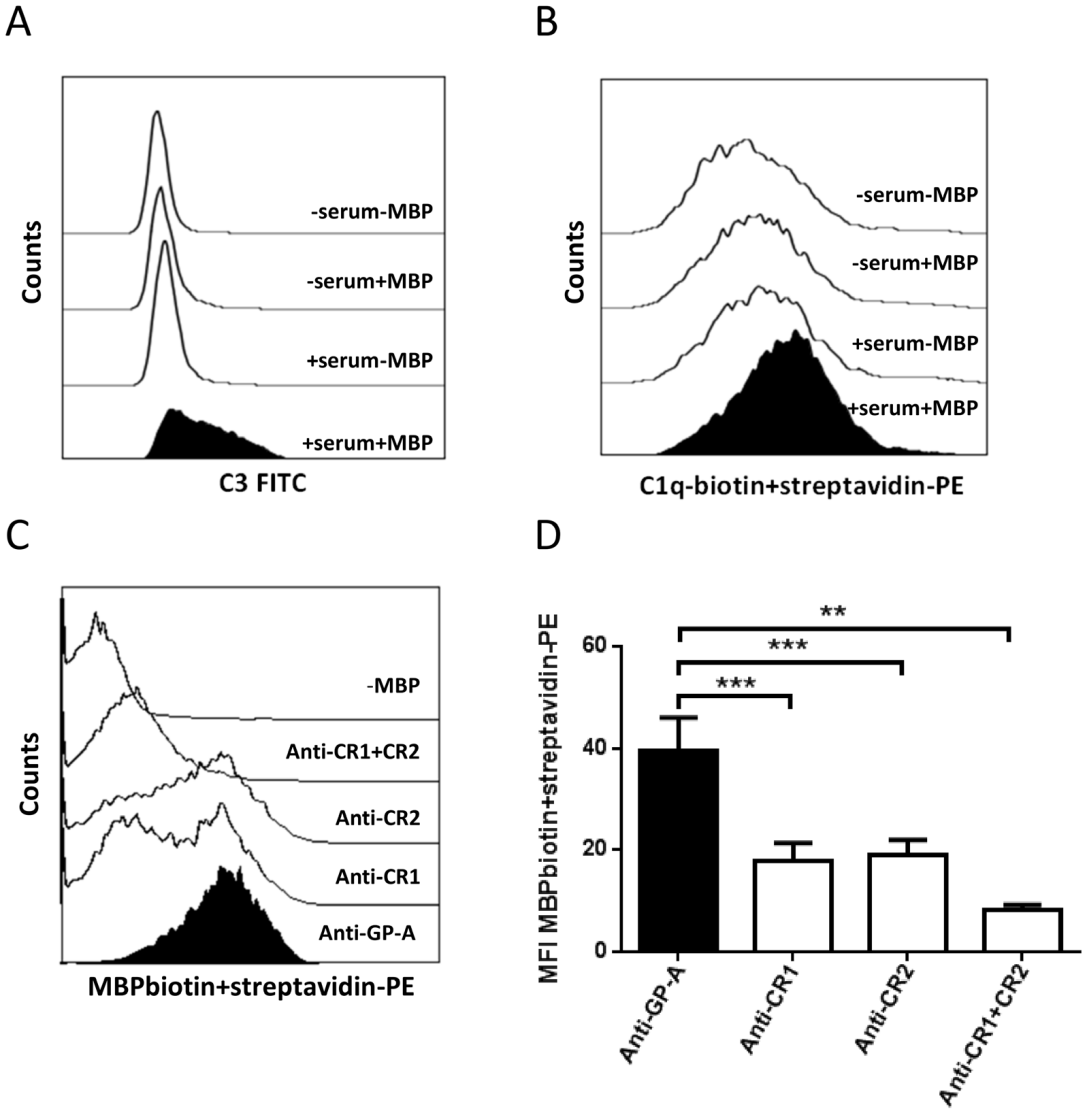
C1q and C3 co-deposit with MBP on B cells, which take up MBP via CR1 and CR2. PBMCs from healthy donors were incubated with or without 30 µg/ml MBP in medium containing normal autologous serum (30% v/v), or in pure medium. The resulting deposition of C3 and C1q on B cells was measured by flow cytometry after 5 min incubation (N = 3). Representative histogram plots show A) C3-deposition, and B) C1q-deposition on B cells. C) The binding of MBP was assessed using biotinylated MBP as probe and subsequent staining with streptavidin-PE. Blockade of CR1 or CR2 was achieved by pre-incubation of PBMCs with mAb3D9 and polyclonal sheep anti-human CR2 respectively. Monoclonal anti-glycophorin (GP)-A was used as negative control. D) Mean fluorescence intensity (MFI) values of 5–6 experiments are shown; background values (of samples with no MBP added) have been subtracted. Bars and error bars represent means and SEM. **p<0.01, ***p<0.001.

In view of previous findings that complement promotes the uptake of antigens by B cells [6], [9], [10], [12], we examined the effect of heating serum to 56°C, which is known to inactivate heat-labile factors of the complement system [35]. Moreover, we also added EDTA or sodium polyanethole sulphonate (SPS) as a different means of preventing complement activation [36], [37]. As shown in Fig. 1C, heat treatment of serum lowered the binding of MBP to B cells by 61.3% on average, while EDTA reduced the binding by 86.1%. SPS reduced the binding of MBP to B cells by 71.6% at a concentration of 0.2 mg/ml and 81.9% at 2.0 mg/ml. Taken together, these data strongly imply that complement enhances the binding of MBP to B cells. Accordingly, we observed that C3-fragments and C1q co-deposited with MBP on the B-cell surface (Figs. 2A and B).

Antibody-mediated blockade of either CR1 or CR2 markedly lowered the binding of MBP to the B cells, while simultaneous blockade of both receptors virtually abrogated MBP binding (Figs. 2C and D). On the contrary, blockade of FcγRIIa,b,c (CD32) known to be expressed by B cells, did not affect the binding of MBP to the B cells (Fig. S1).

### Presentation of the MBP85-99 peptide by normal HLA-DR15+ B cells after culture with whole MBP protein

**Figure 3:**
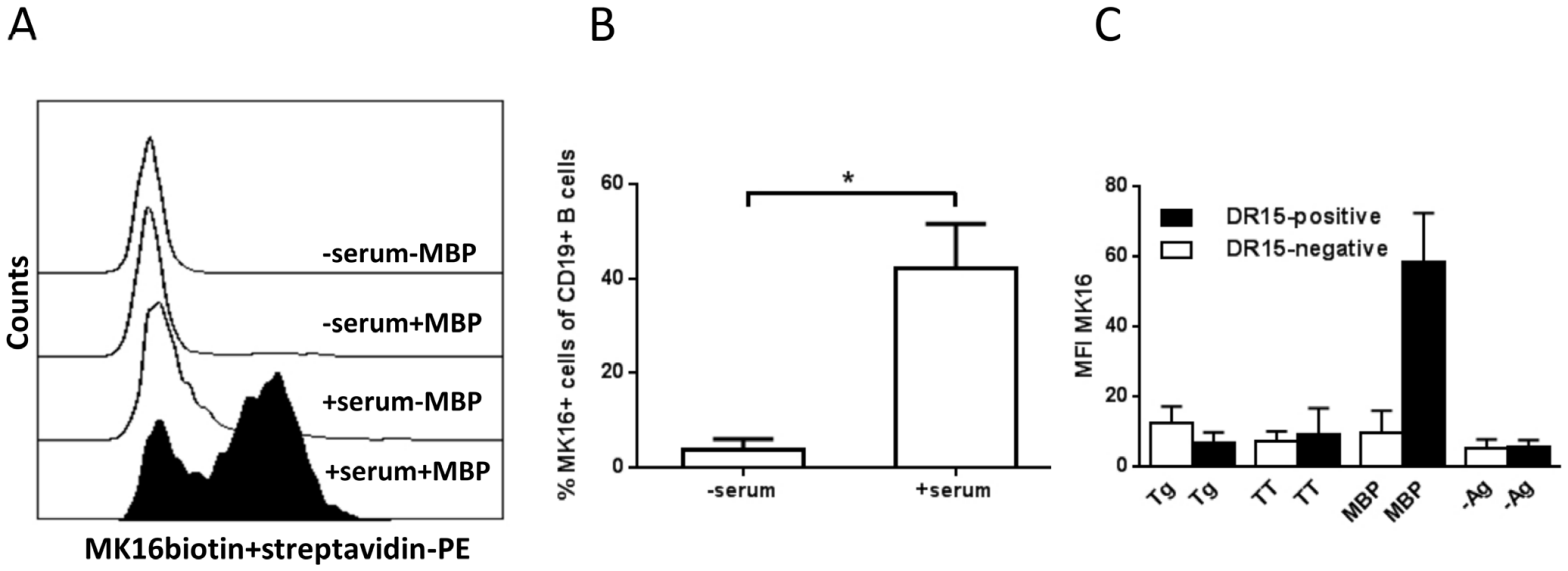
Presentation of MBP85-99 by HLA-DR15+ B cells. PBMCs from HLA-DR15+ individuals were incubated with MBP (whole protein) in the presence or absence of normal serum (30% v/v) for 18 h. Biotinylated mAb MK16 and streptavidin-PE were used as markers of MBP85-99 presentation. A) Representative histogram plot of 5 healthy donors showing binding of MK16 to live (7AAD-negative) B cells in the absence or presence of serum and MPB. B) The percentages of MK16-positive live B cells in 5 healthy HLA-DR15+ donors are shown; background values (no MBP added) have been subtracted. C) MK16 staining of B cells incubated with 30 µg/ml thyroglobulin (Tg), tetanus toxoid (TT), myelin basic protein (MBP) or no antigen (-Ag) in 4 healthy HLA-DR15+ donors (black bars) and 4 healthy HLA-DR15/16 negative donors (white bars). Means and SEM are shown. *p<0.05.

To study antigen presentation by B cells, isolated PBMCs from HLA-DR15-positive donors were incubated with whole MBP. Subsequently, the mAb MK16 was used as probe for presentation of the immunodominant MBP peptide MBP85-99 [28] (Figs. 3A and B). As shown in Fig. 3B, only 3.7±2.4% CD19+ B cells presented MBP peptides in the absence of serum. In the presence of serum, however, 42.2±9.4% of the B-cell population presented MBP85-99. Binding of the MK16 antibody to B cells from DR15-negative donors was also examined to validate the antibody's specificity (Fig. 3C). As expected, MK16 did not bind to MBP-stimulated B cells from DR15-negative subjects, nor to B cells incubated with a different self-antigen, human thyroglobulin, or tetanus toxoid, a foreign recall antigen.

### Influence of serum concentration and complement activity on B-cell presentation of MBP85-99

**Figure 4:**
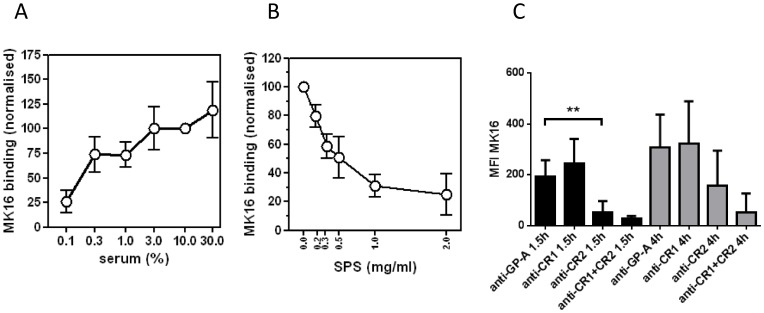
Influence of complement on the presentation of MBP85-99 by DR15+ B cells. PBMCs from healthy HLA-DR15+ donors were incubated for 18 h with MBP in media containing normal serum. Cells were stained with FITC anti-CD19 and biotinylated MK16, followed by streptavidin-PE. (A) The binding of MK16 at different serum concentrations is shown as mean fluorescence (MFI) values normalised to that of 10% serum, (N = 4). B) Before addition of serum (30% v/v), different concentrations of the complement inhibitory compound sodium polyanethole sulphonate (SPS) were added. MFI values are shown, normalised to samples without SPS, (N = 6). (C) The PBMCs were pre-incubated with the anti-CR1 mAb3D9 or polyclonal sheep anti-human CR2, or both, before addition of serum (30% v/v) and MBP. Anti-glycophorin (GP)-A was used as negative control. Data are shown as means±SEM, (N = 4–6). **p<0.01.

Little B-cell presentation of MBP85-99 was observed after incubation of PBMCs with MBP in medium containing only 0.1% of serum (Fig. 4A). At serum concentrations above 3%, however, the peptide was efficiently presented. To examine if complement was the serum factor responsible for enhancing the presentation of MBP85-99, in analogy to its role in binding of MBP by B cells, SPS was used as complement inhibitor and, indeed, dose-dependently reduced the presentation of MBP85-99 (Fig. 4B).

The presence of polyclonal anti-CR2 antibodies during incubation of PBMCs with MBP for 1.5 or 4 h markedly lowered the presentation of MBP85-99 by B cells (Fig. 4C). By contrast, co-incubation with anti-CR1 mAb had no effect (Fig. 4C).

### Phenotypic characterisation of MBP85-99-presenting B cells

**Figure 5:**
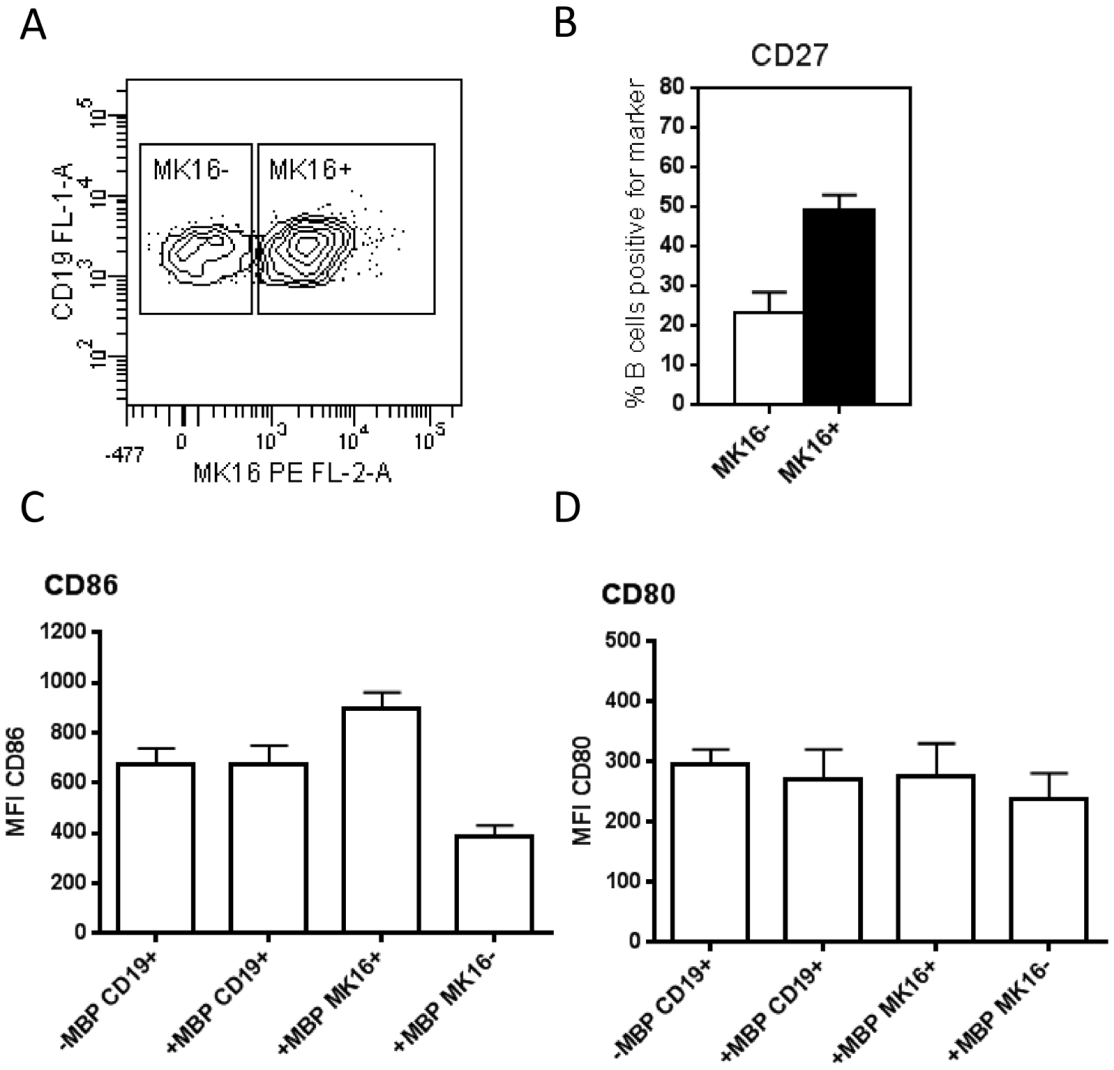
Phenotype of MBP85-99-presenting B cells. PBMCs from healthy HLA-DR15+ donors were incubated with MBP (30 µg/ml) in RPMI containing 30% (v/v) normal serum for 18 h. The presentation of MBP85-99 by CD19+ B cells was assessed using biotinylated MK16 and streptavidin-PE. A) Representative dot plot showing a subset of B cells that present MBP58-99 (MK16+) and a subset that do not (MK16−). Expression of various surface markers was evaluated in these subsets. B) The percentages of MK16+ (black bar) or MK16− (white bar) B cells expressing CD27 are shown (N = 4). B-cell expression of the co-stimulatory molecules C) CD86 and D) CD80 is shown as mean fluorescence intensity (MFI) values (N = 5). 7-AAD was used to exclude dead cells. Data are shown as means and SEM.

To characterise the phenotype of B cells presenting MBP85-99 (Fig. 5A), we co-stained B cells for the expression of the surface markers CD19, CD27, CD5, CD1d, CD24, and IgM.

The most outstanding finding was that a high proportion (around 50%) of B cells presenting MBP85-99 (MK16 positive cells) expressed CD27, which is considered a memory B-cell marker [38], compared to only 20% of the MK16 negative B cells, as shown in Fig. 5B.

Notably, MBP85-99-presenting B cells were not enriched with any of the markers CD5, CD1d, or CD24, which have all been associated with Bregs [20], [23], [39], nor with IgM (Fig. S2). CD86 and CD80 were found to be constitutively expressed by B cells, and their expression was independent on addition of MBP (Figs. 5C and D). Interestingly, however, B cells presenting MBP85-99 showed increased expression of CD86 compared to MBP85-99 negative B cells (Fig. 5C). We did not observe a corresponding increase in the expression of CD80 (Fig. 5D).

In agreement with their lack of phenotypic Breg markers, B cells presenting MBP85-99 with high efficiency did not produce IL-10 or IL-6 when stimulated with MBP alone (Fig. S3).

### Low frequency of IL-10 secreting MBP specific CD4+ T cells in co-cultures of T cells and B cells

**Figure 6:**
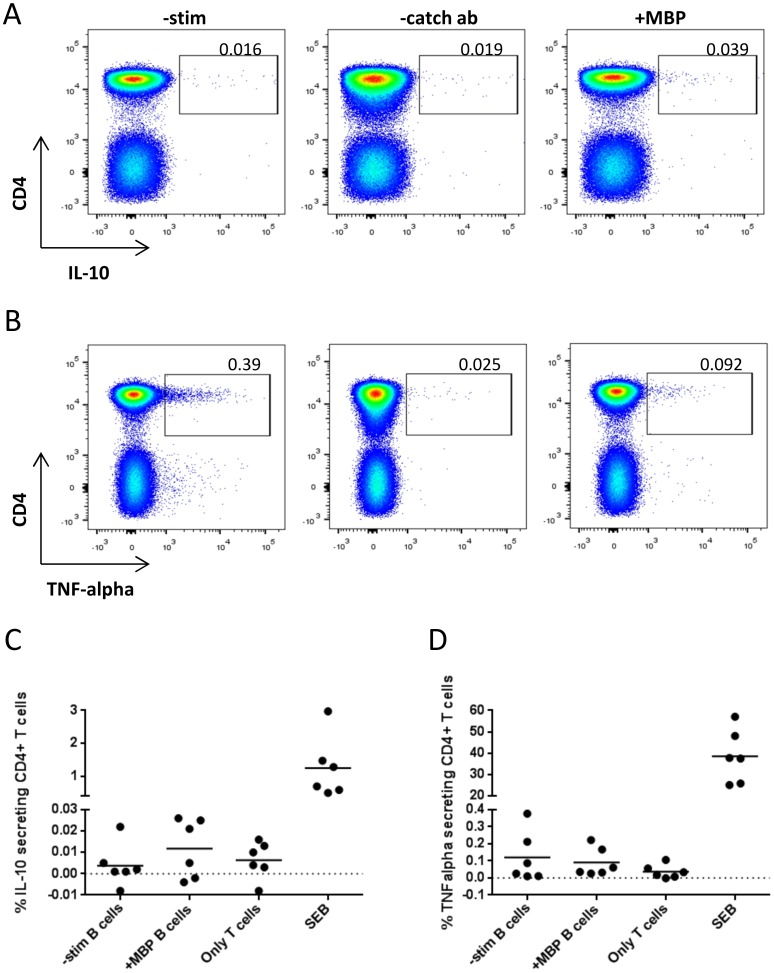
IL-10 and TNF-alpha secretion by MBP specific CD4+ T cells in co-cultures of T cells and B cells. 500,000 CD3+ T cells were cultured with 100,000 CD19+ B cells ±30 µg/ml MBP for 18 h and the resulting IL-10 and TNF-alpha production in CD4+ T cells were measured. Shown is representative dot plots of (A) IL-10 and (B) TNF-alpha production in CD4+ T cells and individual values of the proportion of CD4+ T cells producing (C) IL-10 or (D) TNF-alpha (N = 6). Cultures stimulated by staphylococcal enterotoxin B (SEB) were used as positive controls and cultures containing only T cells were used as negative controls. Samples containing only detection antibodies (-catch ab) were included to correct background staining of the assay and these values were subtracted from all other samples.

In co-cultures of purified CD3+ T cells and purified CD19+ B cells pulsed with MBP, no T-cell proliferation was induced, whereas anti-CD3 stimulated T cells proliferated as expected (data not shown). We did, however, observe a low frequency of IL-10 producing CD4+ T cells in co-cultures of B cells and T cells from three out of six donors, suggesting B cells presenting MBP peptides in some cases drive an immunoregulatory T-cell response (Figs. 6A and C). MBP-pulsed B cells did not induce T-cell production of TNF-alpha in any of the donors tested (Figs. 6B and D).

## Discussion

While the ability of B cells to take up and present foreign antigens has been investigated intensively (for review see [40]), little is known about the capacity of human B cells to take up and present self-antigens and thereby modulate CD4+ T-cell activation. In this study we dissect the uptake and presentation of the self-antigen MBP by B cells.

We observed that 2.5±1.5% of normal B cells were capable of binding MBP when suspended in serum-free medium. We previously showed that a similar proportion of B cells bound a different self-antigen, human thyroglobulin, under similar conditions [12]. It is likely that the B-cell subsets in question bear polyreactive B-cell receptors reactive with a variety of self-and non-self-antigens [41].

In the presence of 30% (v/v) serum, the majority of B cells bound MBP. We show here that B-cell uptake of MBP is dependent on active complement and functional complement receptors. Thus, the binding of MBP was markedly reduced by: i) heat inactivation of serum complement; ii) EDTA-mediated chelation of calcium and magnesium, essential for complement activation; iii) inactivation of complement by SPS [37]; and iv) blockade of CR1 or CR2. Accordingly, we observed that C1q and C3 co-deposited with MBP on B cells. Taken together with the finding that NHS contains antibodies capable of binding MBP [42], [43], this suggests that MBP is incorporated into immune complexes that activate complement via the classic pathway of activation, facilitating the uptake of MBP by B cells as previously shown for exogenous antigens [4]–[6], [9]–[12], [32] and thyroglobulin [12]. We did not detect any lowered binding of MBP when blocking CD32a, b, c indicating that FcγRs do not participate in this process.

When examining the presentation of MBP85-99 peptides after culturing of B cells with whole MBP we found that 3.7±2.4% of B cells presented MBP85-99 in the absence of serum, and that approximately half of the B cells presented the peptide when the serum concentration exceeded 3% (v/v). Thus, a surprisingly high proportion of B cells became engaged in antigen-presentation when serum was present. Inhibition of complement by SPS and antibody-mediated blockade of CR2 abrogated presentation, while anti-CR1 antibody did not inhibit presentation, even though it significantly reduced binding of MBP to B cells. CR2 thus seems to be the important receptor for antigen internalisation by B cells in our system, supporting prior observations [4], [44]. Using a monoclonal antibody recognising the 46–61 determinant from hen egg lysozymes presented on mouse MHC class II molecule I-A^k^ one study has previously demonstrated presentation of peptides by bulk non-specific B cells after administration of soluble protein [45], supporting our findings. The fact that bulk B cells can engage in antigen presentation may have important implications for maintenance of tolerance by Bregs [17], as well as for B-cell-driven pathogenic processes in MS, where pathogenic T cells are believed to be activated outside the blood-brain barrier [46]. Our data suggest that B cells, irrespective of specificity, may take up and present MBP via CR2 in lymph nodes and spleen, or take up MBP outside lymphoid tissue and migrate to secondary lymphoid organs to present MBP-derived peptides to T cells. Transitional B cells may take up MBP outside the blood-brain barrier and, as described by Lee-Chang et al. [31], [47], cross the barrier by virtue of α4 and β1 integrin expression. The majority of MS patients show elevated numbers of B cells in the CNS [31]; B cells have been demonstrated in a subset of cortical lesions in patients with early-stage MS [48], and some patients have ectopic B-cell follicles containing T cells, B cells and plasma cells in the cerebral meninges [49], [50]. Complement-activating immune complexes may also form intrathecally, when anti-MBP antibodies are present in cerebrospinal fluid, as is the case in children at least [42], and immunopathological evidence of complement activation has been demonstrated in plaques [51]. It is possible that higher quantities and affinities of anti-MBP antibodies in patients, as compared to healthy controls, may lead to formation of immune-complexes with stronger pro-inflammatory potential than those involved in this study.

We were not able to identify subsets with particular pro- or anti-inflammatory potential among MBP85-99-presenting B cells, neither in terms of production of IL-10 or IL-6, nor in terms of expression of CD24, CD1d, CD5 or IgM, which have been associated with Bregs [20], [23], [39]. Approximately half of the B cells presenting MBP85-99 expressed CD27, which has been associated with a Breg phenotype [52], [53]. However, it is usually considered a memory B-cell marker [38], hence the significance of this observation is not clear. We did not investigate if the expression of CR1 and CR2 on MBP85-99-presenting B cells was similar to that of B cells not engaged in presentation of the peptide. Others have shown that CD27+ memory B cells have been shown to express higher levels of CR1, but not CR2, than CD27- naïve B cells [54]. Moreover, we observed significantly upregulated expression of CD86 on MBP85-99-presenting B cells. Increased expression of co-stimulatory molecules has usually been associated with an immune activating phenotype of APCs [55], but recently expression of B7 (CD80/CD86) on murine B cells was shown to be central to regulation of CD4+CD25^high^ Tregs in experimental autoimmune encephalomyelitis [18]. Accumulating data also support a role for B cells in the generation of human Tregs [56]. Concordantly, B cells pulsed with MBP induced low frequencies of IL-10-producing CD4+ T cells in half of the co-cultures tested in this study, while MBP induced no TNF-alpha-producing CD4+ T cells. This is to be expected if B cells are involved in the silencing of potentially self-reactive T cells in healthy humans, as indicated by animal experiments [15], [17].

In conclusion, our study demonstrates that B cells, irrespective of specificity, can become engaged in the presentation of the MS-relevant auto-antigen MBP in a complement-dependent manner. While CR1 and CR2 cooperate in the binding of MBP, engagement of CR2 is crucial for subsequent presentation of the immunodominant peptide MBP85-99. Increased expression of CD86 on normal B cells presenting MBP in the presence of NHS indicates a role of these cells in maintenance of tolerance, but different qualities of T cells, B cells and immune complexes in MS may associate the mechanisms described in this study with the pathogenesis of MS.

## Supporting Information

Figure S1 **Contribution of Fcγ-receptors to the binding of MBP to B cells.** PBMCs from three healthy donors were incubated for 30 min. with monoclonal antibodies against FcγRI (anti-CD64, clone10.1), FcγRII (anti-CD32a,b,c, clone AT10) or FcγRIII (anti-CD16, clone 3G8), of which FcγRIIa, -b, and –c are present on mature human B cells, in medium containing 30% (v/v) normal serum. Subsequently, MBP-biotin (30 µg/ml) was added, followed by streptavidin-PE. An antibody against glycophorin-A (anti-GP-A) was used as an additional negative control, and a combination of anti-CR1 antibody (3D9) and polyclonal antibodies against CR2 were included as positive controls for inhibition. The binding of MBP-biotin/streptavidin-PE was assessed by flow cytometry. The resulting mean fluorescence intensity (MFI) values are shown as mean±SEM.
(TIF)

Figure S2 **Phenotype of MBP85-99-presenting B cells.** PBMCs from four healthy HLA-DR15+ donors were incubated with MBP (30 µg/ml) in RPMI medium containing 30% (v/v) normal serum. The presentation of MBP85-99 by CD19+ B cells was assessed by flow cytometry using FITC-conjugated MK16 antibody (A) or biotinylated MK16+streptavidin-PE (C–D). Shown is the percentage of MK16- and MK16+ B cells expressing CD5 (A), CD24 (B), CD1d (C), or IgM (D) among B cells. MFI values are shown as mean±SEM.
(TIF)

Figure S3 **Cytokine secretion by MBP85-99 presenting B cells.** PBMCs from four healthy HLA-DR15+ donors were incubated for 18 hours with or without MBP (30 µg/ml) in RPMI medium containing 30% (v/v) normal serum. Cells were stained with PerCP anti-human CD19, biotinylated MK16+PE-streptavidin, APC-anti-human IL-10, FITC anti-human IL-6 and life/dead cell discriminator LIVE/DEAD Fixable Near-IR. A) Representative dot plot showing IL-10 and IL-6 secretion by MBP85-99 presenting, live B cells. B) The percentages of IL-10 producing or C) IL-6 producing, live B cells are shown as means and SEM. As positive control, a combination of MBP, phorbol myristate acetate and ionomycin (PMAiono) was used as stimulating agent.
(TIF)

## References

[1] Pistoia V (1997) Production of cytokines by human B cells in health and disease. Immunol Today 18: 343–350. 9238838 10.1016/s0167-5699(97)01080-3

[2] Lanzavecchia A (1985) Antigen-specific interaction between T and B cells. Nature 314: 537–539. 3157869 10.1038/314537a0

[3] Carroll MC (1998) The role of complement and complement receptors in induction and regulation of immunity. Annu Rev Immunol 16: 545–568. 9597141 10.1146/annurev.immunol.16.1.545

[4] Barrault DV, Knight AM (2004) Distinct sequences in the cytoplasmic domain of complement receptor 2 are involved in antigen internalization and presentation. J Immunol 172: 3509–3517. 15004151 10.4049/jimmunol.172.6.3509

[5] Arvieux J, Yssel H, Colomb MG (1988) Antigen-bound C3b and C4b enhance antigen-presenting cell function in activation of human T-cell clones. Immunology 65: 229–235. 2973431 PMC1384918

[6] Boackle SA, Morris MA, Holers VM, Karp DR (1998) Complement opsonization is required for presentation of immune complexes by resting peripheral blood B cells. J Immunol 161: 6537–6543. 9862679

[7] Nielsen CH, Matthiesen SH, Lyng I, Leslie RG (1997) The role of complement receptor type 1 (CR1, CD35) in determining the cellular distribution of opsonized immune complexes between whole blood cells: kinetic analysis of the buffering capacity of erythrocytes. Immunology 90: 129–137. 9038723 10.1046/j.1365-2567.1997.00138.x PMC1456720

[8] Nielsen CH, Svehag SE, Marquart HV, Leslie RG (1994) Interactions of opsonized immune complexes with whole blood cells: binding to erythrocytes restricts complex uptake by leucocyte populations. Scand J Immunol 40: 228–236. 8047846 10.1111/j.1365-3083.1994.tb03455.x

[9] Thornton BP, Vetvicka V, Ross GD (1996) Function of C3 in a humoral response: iC3b/C3dg bound to an immune complex generated with natural antibody and a primary antigen promotes antigen uptake and the expression of co-stimulatory molecules by all B cells, but only stimulates immunoglobulin synthesis by antigen-specific B cells. Clin Exp Immunol 104: 531–537. 9099940 10.1046/j.1365-2249.1996.57761.x PMC2200445

[10] Thornton BP, Vetvicka V, Ross GD (1994) Natural antibody and complement-mediated antigen processing and presentation by B lymphocytes. J Immunol 152: 1727–1737. 8120381

[11] Villiers MB, Villiers CL, Jacquier-Sarlin MR, Gabert FM, Journet AM, et al (1996) Covalent binding of C3b to tetanus toxin: influence on uptake/internalization of antigen by antigen-specific and non-specific B cells. Immunology 89: 348–355. 8958046 10.1046/j.1365-2567.1996.d01-747.x PMC1456555

[12] Nielsen CH, Leslie RG, Jepsen BS, Kazatchkine MD, Kaveri SV, et al (2001) Natural autoantibodies and complement promote the uptake of a self antigen, human thyroglobulin, by B cells and the proliferation of thyroglobulin-reactive CD4(+) T cells in healthy individuals. Eur J Immunol 31: 2660–2668. 11536164 10.1002/1521-4141(200109)31:9<2660::aid-immu2660>3.0.co;2-e

[13] Nielsen CH, Hegedus L, Leslie RG (2004) Autoantibodies in autoimmune thyroid disease promote immune complex formation with self antigens and increase B cell and CD4+ T cell proliferation in response to self antigens. Eur J Immunol 34: 263–272. 14971052 10.1002/eji.200324413

[14] Mauri C, Bosma A (2012) Immune regulatory function of B cells. Annu Rev Immunol 30: 221–241. 22224776 10.1146/annurev-immunol-020711-074934

[15] Mauri C, Gray D, Mushtaq N, Londei M (2003) Prevention of arthritis by interleukin 10-producing B cells. J Exp Med 197: 489–501. 12591906 10.1084/jem.20021293 PMC2193864

[16] O'Garra A, Howard M (1992) IL-10 production by CD5 B cells. Ann N Y Acad Sci 651: 182–199. 1376039 10.1111/j.1749-6632.1992.tb24615.x

[17] Fillatreau S, Sweenie CH, McGeachy MJ, Gray D, Anderton SM (2002) B cells regulate autoimmunity by provision of IL-10. Nat Immunol 3: 944–950. 12244307 10.1038/ni833

[18] Mann MK, Maresz K, Shriver LP, Tan Y, Dittel BN (2007) B cell regulation of CD4+CD25+ T regulatory cells and IL-10 via B7 is essential for recovery from experimental autoimmune encephalomyelitis. J Immunol 178: 3447–3456. 17339439 10.4049/jimmunol.178.6.3447

[19] Carter NA, Vasconcellos R, Rosser EC, Tulone C, Munoz-Suano A, et al (2011) Mice lacking endogenous IL-10-producing regulatory B cells develop exacerbated disease and present with an increased frequency of Th1/Th17 but a decrease in regulatory T cells. J Immunol 186: 5569–5579. 21464089 10.4049/jimmunol.1100284

[20] Blair PA, Norena LY, Flores-Borja F, Rawlings DJ, Isenberg DA, et al (2010) CD19(+)CD24(hi)CD38(hi) B cells exhibit regulatory capacity in healthy individuals but are functionally impaired in systemic Lupus Erythematosus patients. Immunity 32: 129–140. 20079667 10.1016/j.immuni.2009.11.009

[21] Duddy M, Niino M, Adatia F, Hebert S, Freedman M, et al (2007) Distinct effector cytokine profiles of memory and naive human B cell subsets and implication in multiple sclerosis. J Immunol 178: 6092–6099. 17475834 10.4049/jimmunol.178.10.6092

[22] Fillatreau S, Gray D, Anderton SM (2008) Not always the bad guys: B cells as regulators of autoimmune pathology. Nat Rev Immunol 8: 391–397. 18437156 10.1038/nri2315

[23] Langkjaer A, Kristensen B, Hansen BE, Schultz H, Hegedus L, et al (2012) B-cell exposure to self-antigen induces IL-10 producing B cells as well as IL-6- and TNF-alpha-producing B-cell subsets in healthy humans. Clin Immunol 145: 1–10. 22885146 10.1016/j.clim.2012.07.004

[24] Nielsen CH, El FD, Hasselbalch HC, Bendtzen K, Hegedus L (2007) B-cell depletion with rituximab in the treatment of autoimmune diseases. Graves' ophthalmopathy the latest addition to an expanding family. Expert Opin Biol Ther 7: 1061–1078. 17665994 10.1517/14712598.7.7.1061

[25] Hauser SL, Waubant E, Arnold DL, Vollmer T, Antel J, et al (2008) B-cell depletion with rituximab in relapsing-remitting multiple sclerosis. N Engl J Med 358: 676–688. 18272891 10.1056/NEJMoa0706383

[26] Bar-Or A, Calabresi PA, Arnold D, Markowitz C, Shafer S, et al (2008) Rituximab in relapsing-remitting multiple sclerosis: a 72-week, open-label, phase I trial. Ann Neurol 63: 395–400. 18383069 10.1002/ana.21363

[27] Sospedra M, Martin R (2005) Immunology of multiple sclerosis. Annu Rev Immunol 23: 683–747. 15771584 10.1146/annurev.immunol.23.021704.115707

[28] Krogsgaard M, Wucherpfennig KW, Cannella B, Hansen BE, Svejgaard A, et al (2000) Visualization of myelin basic protein (MBP) T cell epitopes in multiple sclerosis lesions using a monoclonal antibody specific for the human histocompatibility leukocyte antigen (HLA)-DR2-MBP 85-99 complex. J Exp Med 191: 1395–1412. 10770805 10.1084/jem.191.8.1395 PMC2193136

[29] Weber MS, Prod'homme T, Patarroyo JC, Molnarfi N, Karnezis T, et al (2010) B-cell activation influences T-cell polarization and outcome of anti-CD20 B-cell depletion in central nervous system autoimmunity. Ann Neurol 68: 369–383. 20641064 10.1002/ana.22081 PMC3375897

[30] Barr TA, Shen P, Brown S, Lampropoulou V, Roch T, et al (2012) B cell depletion therapy ameliorates autoimmune disease through ablation of IL-6-producing B cells. J Exp Med 209: 1001–1010. 22547654 10.1084/jem.20111675 PMC3348102

[31] Cepok S, Rosche B, Grummel V, Vogel F, Zhou D, et al (2005) Short-lived plasma blasts are the main B cell effector subset during the course of multiple sclerosis. Brain 128: 1667–1676. 15800022 10.1093/brain/awh486

[32] Jacquier-Sarlin MR, Gabert FM, Villiers MB, Colomb MG (1995) Modulation of antigen processing and presentation by covalently linked complement C3b fragment. Immunology 84: 164–170. 7890301 PMC1415195

[33] Ciechomska M, Wilson CL, Floudas A, Hui W, Rowan AD, et al (2014) Antigen-specific B lymphocytes acquire proteoglycan aggrecan from cartilage extracellular matrix resulting in antigen presentation and CD4+ T-cell activation. Immunology 141: 70–78. 24032649 10.1111/imm.12169 PMC3893851

[34] Jensen LB, Riise E, Nielsen LK, Dziegiel M, Fugger L, et al (2004) Efficient purification of unique antibodies using peptide affinity-matrix columns. J Immunol Methods 284: 45–54. 14736416 10.1016/j.jim.2003.10.001

[35] Johnson AH, Mowbray JF, Porter KA (1975) Detection of circulating immune complexes in pathological human sera. Lancet 1: 762–765. 47998 10.1016/s0140-6736(75)92433-2

[36] Nielsen CH, Pedersen ML, Marquart HV, Prodinger WM, Leslie RG (2002) The role of complement receptors type 1 (CR1, CD35) and 2 (CR2, CD21) in promoting C3 fragment deposition and membrane attack complex formation on normal peripheral human B cells. Eur J Immunol 32: 1359–1367. 11981823 10.1002/1521-4141(200205)32:5<1359::AID-IMMU1359>3.0.CO;2-V

[37] Palarasah Y, Skjoedt MO, Vitved L, Andersen TE, Skjoedt K, et al (2010) Sodium polyanethole sulfonate as an inhibitor of activation of complement function in blood culture systems. J Clin Microbiol 48: 908–914. 20042630 10.1128/JCM.01985-09 PMC2832435

[38] Agematsu K, Hokibara S, Nagumo H, Komiyama A (2000) CD27: a memory B-cell marker. Immunol Today 21: 204–206. 10782048 10.1016/s0167-5699(00)01605-4

[39] Yanaba K, Bouaziz JD, Haas KM, Poe JC, Fujimoto M, et al (2008) A regulatory B cell subset with a unique CD1dhiCD5+ phenotype controls T cell-dependent inflammatory responses. Immunity 28: 639–650. 18482568 10.1016/j.immuni.2008.03.017

[40] Lanzavecchia A (1990) Receptor-mediated antigen uptake and its effect on antigen presentation to class II-restricted T lymphocytes. Annu Rev Immunol 8: 773–793. 2188679 10.1146/annurev.iy.08.040190.004013

[41] Nakamura M, Burastero SE, Ueki Y, Larrick JW, Notkins AL, et al (1988) Probing the normal and autoimmune B cell repertoire with Epstein-Barr virus. Frequency of B cells producing monoreactive high affinity autoantibodies in patients with Hashimoto's disease and systemic lupus erythematosus. J Immunol 141: 4165–4172. 2848890

[42] O'Connor KC, Lopez-Amaya C, Gagne D, Lovato L, Moore-Odom NH, et al (2010) Anti-myelin antibodies modulate clinical expression of childhood multiple sclerosis. J Neuroimmunol 223: 92–99. 20381173 10.1016/j.jneuroim.2010.02.019

[43] O'Connor KC, Chitnis T, Griffin DE, Piyasirisilp S, Bar-Or A, et al (2003) Myelin basic protein-reactive autoantibodies in the serum and cerebrospinal fluid of multiple sclerosis patients are characterized by low-affinity interactions. J Neuroimmunol 136: 140–148. 12620653 10.1016/s0165-5728(03)00002-x

[44] Boackle SA, Holers VM, Karp DR (1997) CD21 augments antigen presentation in immune individuals. Eur J Immunol 27: 122–129. 9022008 10.1002/eji.1830270119

[45] Zhong G (1997) Reis e Sousa (1997) Germain RN (1997) Antigen-unspecific B cells and lymphoid dendritic cells both show extensive surface expression of processed antigen-major histocompatibility complex class II complexes after soluble protein exposure in vivo or in vitro. J Exp Med 186: 673–682. 9271583 10.1084/jem.186.5.673 PMC2199022

[46] Goverman J (2009) Autoimmune T cell responses in the central nervous system. Nat Rev Immunol 9: 393–407. 19444307 10.1038/nri2550 PMC2813731

[47] Lee-Chang C, Top I, Zephir H, Dubucquoi S, Trauet J, et al (2011) Primed status of transitional B cells associated with their presence in the cerebrospinal fluid in early phases of multiple sclerosis. Clin Immunol 139: 12–20. 21310664 10.1016/j.clim.2010.11.004

[48] Lucchinetti CF, Popescu BF, Bunyan RF, Moll NM, Roemer SF, et al (2011) et al Inflammatory cortical demyelination in early multiple sclerosis. N Engl J Med 365: 2188–2197. 22150037 10.1056/NEJMoa1100648 PMC3282172

[49] Magliozzi R, Howell O, Vora A, Serafini B, Nicholas R, et al (2007) Meningeal B-cell follicles in secondary progressive multiple sclerosis associate with early onset of disease and severe cortical pathology. Brain 130: 1089–1104. 17438020 10.1093/brain/awm038

[50] Serafini B, Rosicarelli B, Magliozzi R, Stigliano E, Aloisi F (2004) Detection of ectopic B-cell follicles with germinal centers in the meninges of patients with secondary progressive multiple sclerosis. Brain Pathol 14: 164–174. 15193029 10.1111/j.1750-3639.2004.tb00049.x PMC8095922

[51] Lucchinetti C, Bruck W, Parisi J, Scheithauer B, Rodriguez M, et al (2000) Heterogeneity of multiple sclerosis lesions: implications for the pathogenesis of demyelination. Ann Neurol 47: 707–717. 10852536 10.1002/1531-8249(200006)47:6<707::aid-ana3>3.0.co;2-q

[52] Iwata Y, Matsushita T, Horikawa M, Dilillo DJ, Yanaba K, et al (2011) Characterization of a rare IL-10-competent B-cell subset in humans that parallels mouse regulatory B10 cells. Blood 117: 530–541. 20962324 10.1182/blood-2010-07-294249 PMC3031478

[53] Bouaziz JD, Calbo S, Maho-Vaillant M, Saussine A, Bagot M, et al (2010) IL-10 produced by activated human B cells regulates CD4(+) T-cell activation in vitro. Eur J Immunol 40: 2686–2691. 20809522 10.1002/eji.201040673

[54] Isaak A, Gergely P Jr, Szekeres Z, Prechl J, Poor G, et al (2008) Physiological up-regulation of inhibitory receptors Fc gamma RII and CR1 on memory B cells is lacking in SLE patients. Int Immunol 20: 185–192. 18182380 10.1093/intimm/dxm132

[55] Lenschow DJ, Walunas TL, Bluestone JA (1996) CD28/B7 system of T cell costimulation. Annu Rev Immunol 14: 233–258. 8717514 10.1146/annurev.immunol.14.1.233

[56] Lund FE, Randall TD (2010) Effector and regulatory B cells: modulators of CD4+ T cell immunity. Nat Rev Immunol 10: 236–247. 20224569 10.1038/nri2729 PMC3038334

