# Supplementary figures and images for: Uptake and Presentation of Myelin Basic Protein by Normal Human B Cells
Source: PLoS One. 2014 Nov 17;9(11):e113388. doi: 10.1371/journal.pone.0113388 (PMC4234674; doi:10.1371/journal.pone.0113388)

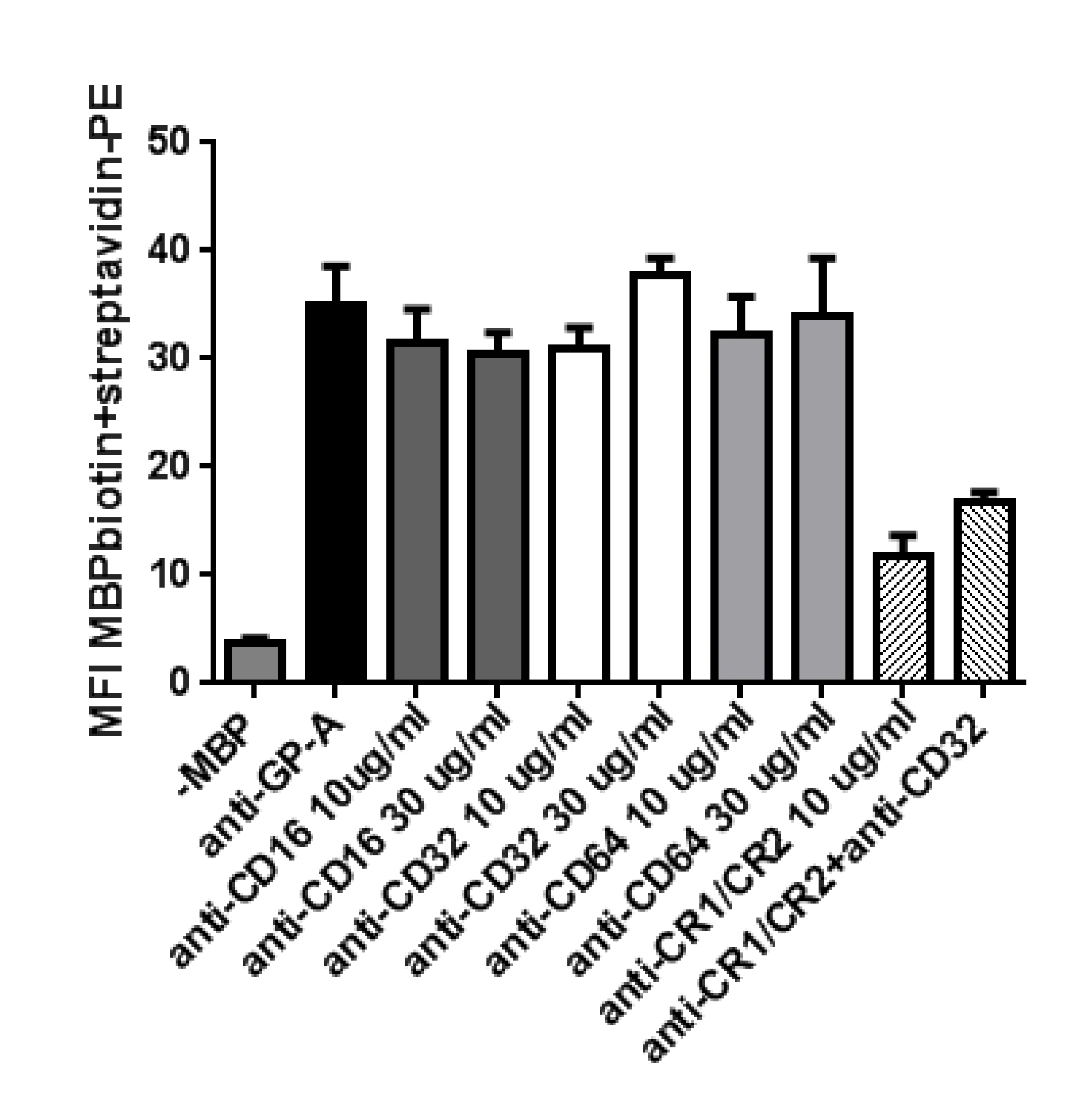

Supplement: Figure S1 — Contribution of Fcγ-receptors to the binding of MBP to B cells. PBMCs from three healthy donors were incubated for 30 min. with monoclonal antibodies against FcγRI (anti-CD64, clone10.1), FcγRII (anti-CD32a,b,c, clone AT10) or FcγRIII (anti-CD16, clone 3G8), of which FcγRIIa, -b, and –c are present on mature human B cells, in medium containing 30% (v/v) normal serum. Subsequently, MBP-biotin (30 µg/ml) was added, followed by streptavidin-PE. An antibody against glycophorin-A (anti-GP-A) was used as an additional negative control, and a combination of anti-CR1 antibody (3D9) and polyclonal antibodies against CR2 were included as positive controls for inhibition. The binding of MBP-biotin/streptavidin-PE was assessed by flow cytometry. The resulting mean fluorescence intensity (MFI) values are shown as mean±SEM. (TIF) [file pone.0113388.s001.tif]

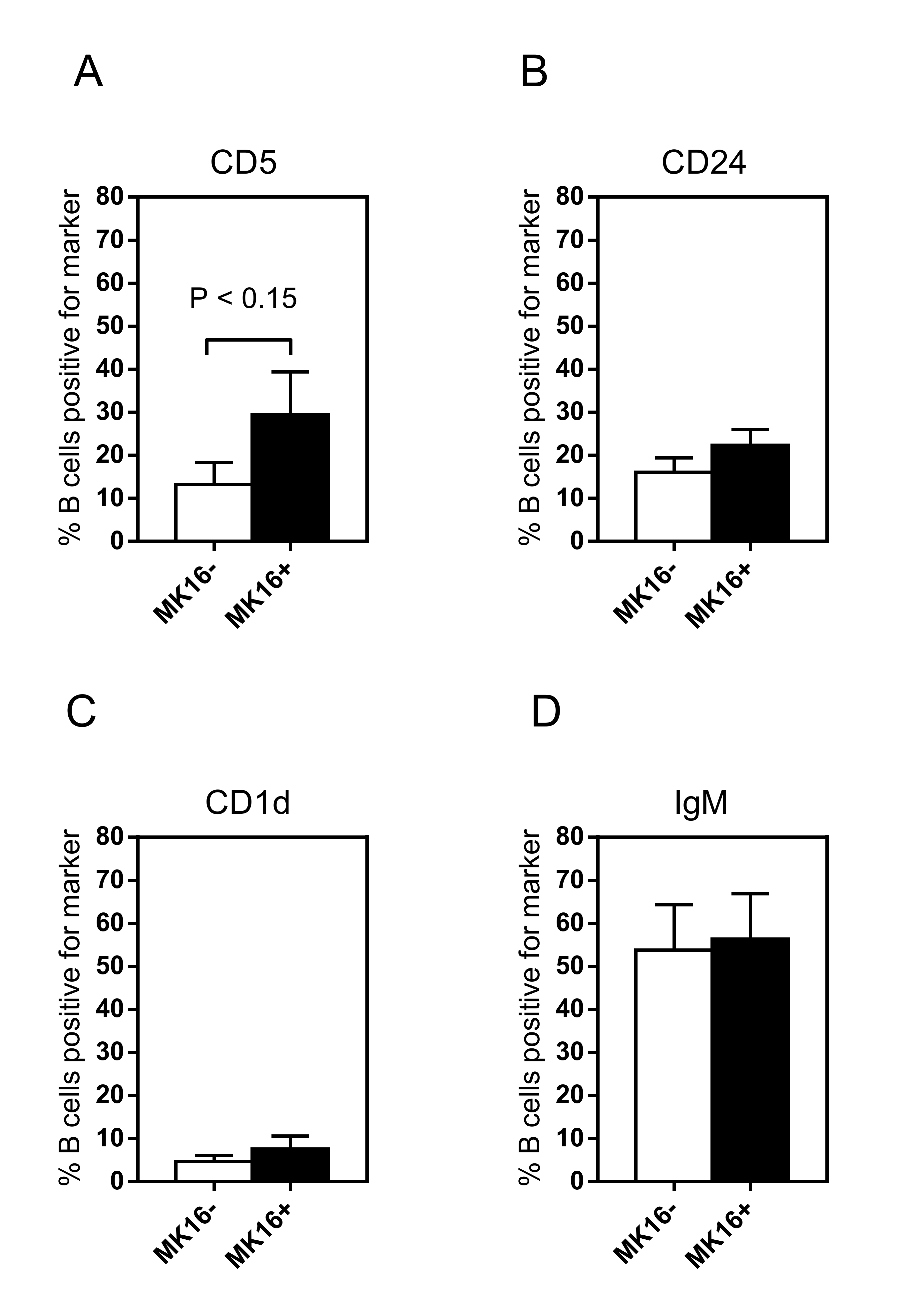

Supplement: Figure S2 — Phenotype of MBP85-99-presenting B cells. PBMCs from four healthy HLA-DR15+ donors were incubated with MBP (30 µg/ml) in RPMI medium containing 30% (v/v) normal serum. The presentation of MBP85-99 by CD19+ B cells was assessed by flow cytometry using FITC-conjugated MK16 antibody (A) or biotinylated MK16+streptavidin-PE (C–D). Shown is the percentage of MK16- and MK16+ B cells expressing CD5 (A), CD24 (B), CD1d (C), or IgM (D) among B cells. MFI values are shown as mean±SEM. (TIF) [file pone.0113388.s002.tif]

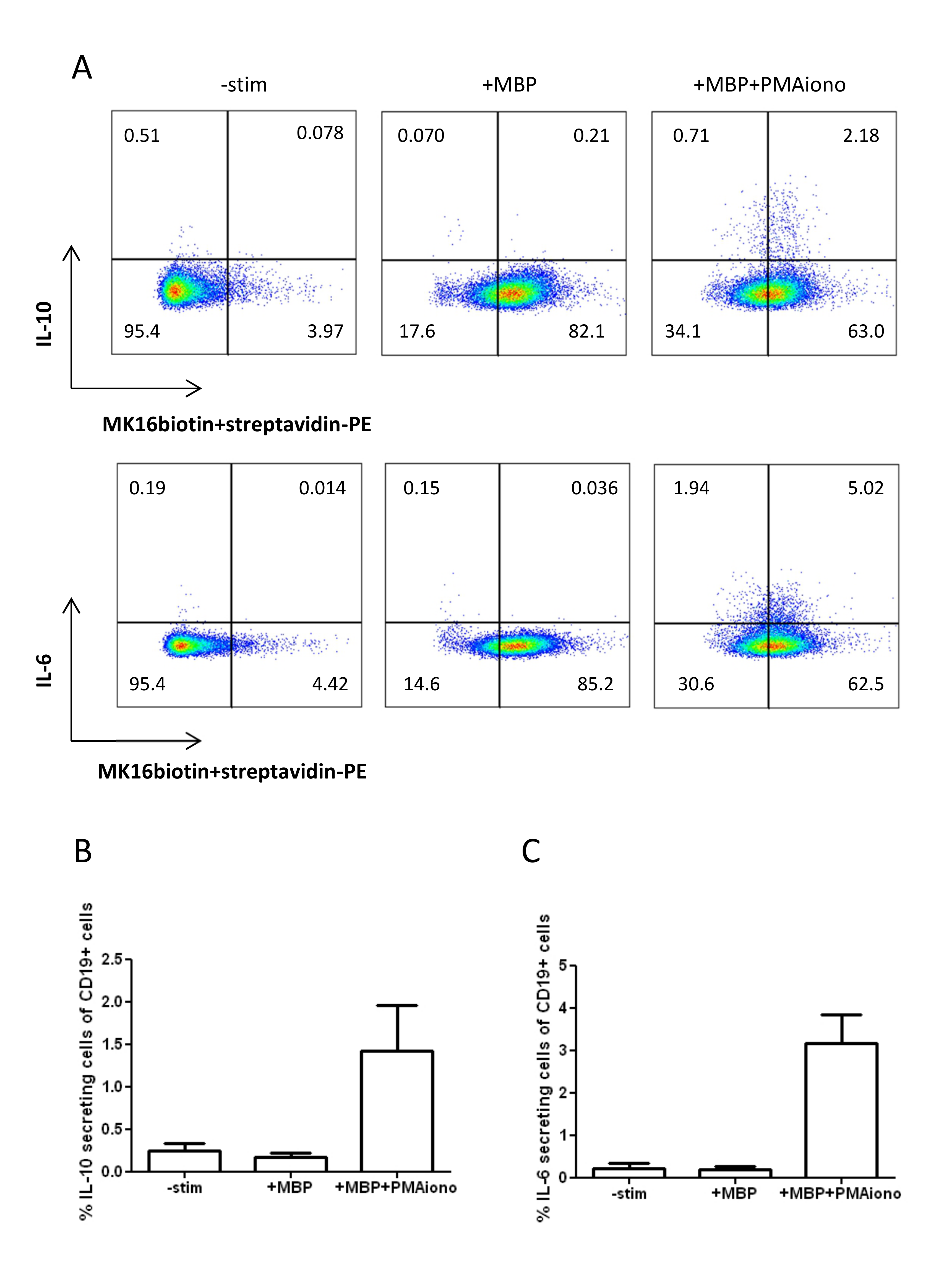

Supplement: Figure S3 — Cytokine secretion by MBP85-99 presenting B cells. PBMCs from four healthy HLA-DR15+ donors were incubated for 18 hours with or without MBP (30 µg/ml) in RPMI medium containing 30% (v/v) normal serum. Cells were stained with PerCP anti-human CD19, biotinylated MK16+PE-streptavidin, APC-anti-human IL-10, FITC anti-human IL-6 and life/dead cell discriminator LIVE/DEAD Fixable Near-IR. A) Representative dot plot showing IL-10 and IL-6 secretion by MBP85-99 presenting, live B cells. B) The percentages of IL-10 producing or C) IL-6 producing, live B cells are shown as means and SEM. As positive control, a combination of MBP, phorbol myristate acetate and ionomycin (PMAiono) was used as stimulating agent. (TIF) [file pone.0113388.s003.tif]
